# Systematic selection of small molecules to promote differentiation of embryonic stem cells and experimental validation for generating cardiomyocytes

**DOI:** 10.1038/cddiscovery.2016.7

**Published:** 2016-02-08

**Authors:** Y KalantarMotamedi, M Peymani, H Baharvand, M H Nasr-Esfahani, A Bender

**Affiliations:** 1 Centre for Molecular Informatics, Department of Chemistry, University of Cambridge, Lensfield Road, Cambridge CB2 1EW, UK; 2 Department of Cell and Molecular Biology, Cell Science Research Centre, Royan Institute for Biotechnology, ACECR, Isfahan, Iran; 3 Department of Stem Cells and Developmental Biology, Cell Science Research Center, Royan Institute for Stem Cell Biology and Technology, ACECR, Tehran, Iran

## Abstract

Small molecules are being increasingly used for inducing the targeted differentiation of stem cells to different cell types. However, until now no systematic method for selecting suitable small molecules for this purpose has been presented. In this work, we propose an integrated and general bioinformatics- and cheminformatics-based approach for selecting small molecules which direct cellular differentiation in the desired way. The approach was successfully experimentally validated for differentiating stem cells into cardiomyocytes. All predicted compounds enhanced expression of cardiac progenitor (Gata4, Nkx2-5 and Mef2c) and mature cardiac markers (Actc1, myh6) significantly during and post-cardiac progenitor formation. The best-performing compound, Famotidine, increased the percentage of Myh6-positive cells from 33 to 56%, and enhanced the expression of Nkx2.5 and Tnnt2 cardiac progenitor and cardiac markers in protein level. The approach employed in the study is applicable to all other stem cell differentiation settings where gene expression data are available.

## Introduction

Differentiating stem cells to different tissues is of current major and increasing importance in the context of regenerative medicine. Transcription factors have been used for inducing the differentiation of embryonic stem cells in a step-wise manner to various cells of interest such as dopamine neurons, retinal pigment epithelium, floor plate cells, hematopoietic cells, endothelial cells, pancreatic cells and cardiomyocytes.^1^ However, the utilization of transcription factor still suffers from shortcomings such as reproducibility, efficiency, cost and quality (eg, homogeneous differentiation) which prevents translation of these methods into therapy and clinic.^1,2^ Hence, the utilization of small molecules is often preferred as it is safer, more efficient, more robust and more cost effective.^1,3,4^ Various small molecules have been identified that can induce the differentiation of stem cells to different tissues.^1,4,5^ Selecting small molecules for the differentiation of stem cells to cardiomyocytes is of particular interest^6–9^ because this cell type can be used as a valuable cell source for replacement therapy following myocardial injury, as well as being able to serve as a cardiovascular disease model for drug screening.^10^ However, to the best of our knowledge, currently there exists no systematic approach to facilitate the general and data-driven selection of small molecules for the differentiation of stem cells.

**Figure 1:**
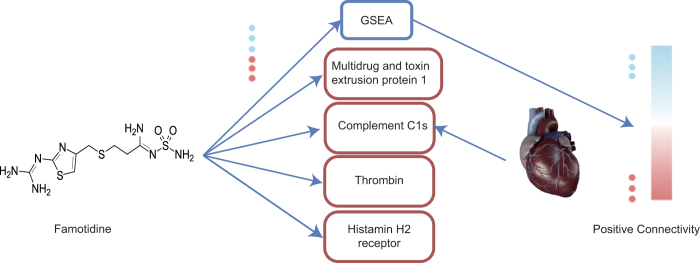
Integrated bioinformatics and cheminformatics approach for selecting compounds for cardiomyocyte differentiation. Both gene expression data (blue and red dots, for up- and downregulation, respectively) and target predictions (proteins in red boxes) are taken into account in the approach presented here. The bioinformatics component was able to detect strong connectivity between the Famotidine gene signature and the heart gene expression profile (Embryonic stem cells *versus* adult heart). The cheminformatics approach on the other hand, specifies potential established (Histamine H2) and novel targets of Famotidine. CTD suggests that Complement C1s is important protein in cardiovascular diseases. Hence, the combination of ligand-target associations and gene expression data are able to provide unified view for guiding compound selection, and understanding its activity in a biological system.

Hence, in this work we present a systematic approach for the selection of potent small molecules for inducing the differentiation of pluripotent stem cells to the tissue of interest. We have applied and experimentally validated this approach, which is based both on bioinformatics and cheminformatics components, by selecting small molecules to promote the differentiation of stem cells to cardiomyocytes. The approach presented here employs publicly available gene expression data for the transition from stem cells to cardiomyocytes from the cellular side, and gene expression data that are the result of compound treatment from the other side. On the basis of matching changes in gene expression in both spaces (upon compound treatment, as well as upon differentiation) our algorithm predicts candidate compounds to induce the differentiation of stem cells to cardiomyocytes (Figure 1). The gene expression database that includes both cardiomyocytes and embryonic stem cell samples have been selected from Gene Expression Omnibus (GEO),^11^ while gene expression data for 1309 compound treatments has been employed as provided in the Connectivity Map (CMap)^12^ database. The CMap database has been previously used particularly in drug repositioning and also predicting the mode-of-action of drugs,^13,14^ however, its use in the context of cellular differentiation is novel.

In the area of drug repositioning it is hypothesized that if the gene expression signature of compound treatment is strongly anticorrelated to a given disease signature (ie, behaves in the precisely opposite way), then that compound is potentially capable of treating this disease.^15^ Experimental evidence supporting this hypothesis has been presented in several studies, usually also based on the CMap database,^13,14^ such as the repurposing of the antiulcer drug Cimetidine for lung cancer^15^ and antiepileptic Topiramate for inflammatory bowel disease.^15^ On the basis of the same hypothesis, the authors have recently developed transcriptional approaches for repurposing drugs for various diseases with prospective successful validation for leukemia and breast cancer.^16^

In this work, we have been inspired by the idea of matching gene signatures of a disease to drugs and applied it into novel application area of stem cell differentiation. Although in drug repositioning disease and healthy samples are compared with constitute the gene expression profile of a disease, in the case of selecting small molecules for stem cell differentiation adult heart samples are compared with embryonic stem cells to constitute the heart gene expression profile. Subsequently, the gene expression profile of this tissue is matched with compound signatures using Gene Set Enrichment Analysis (GSEA).^17^ The GSEA approach yields in connectivity scores that represents how much the genes targeted by each compound is related to genes differentially expressed in the heart signature where correlated, anticorrelated and not correlated gene signatures (between compound and heart signature) results in positive connectivity, negative connectivity and zero connectivity scores.

In addition to this gene expression-based ranking of compounds for a particular purpose we also took its computationally predicted bioactivity spectra (its predicted activities against a set of 1080 protein targets) into account. For this purpose a cheminformatics target prediction algorithm^18^ that was previously developed was extended (see Experimental Procedures) and all the putative protein targets of the compounds contained in CMap were predicted. In addition, for the purpose of the study here all the predicted protein targets in cardiovascular diseases using the Comparative Toxicogenomics Database (CTD)^19^ were determined. The aim of this step was to enhance the compound selection process by giving insights to the potential targets of the compounds and thereby providing a clear mechanistic hypothesis for the mode-of-action of the compounds selected for differentiation.

Subsequently, the combined bioinformatics and cheminformatics approach presented here was employed in the novel area of systematic selection of small molecules for promoting differentiation of stem cell to cardiomyocytes. It was observed that administration of four novel-predicted compounds significantly enhance expansion rate and expression of cardiac precursor and cardiac markers in gene and protein level. This method is generally applicable in cases where gene expression data of the target tissue are available.

## Results

The expression profile of undifferentiated human embryonic stem cells was compared with the expression profile of adult ventricular cardiac tissue using database GSE50704 in GEO (see Supplementary Table S1 for the full list of rank-ordered compounds with negative and positive connectivity). In order to retrospectively validate the method, we firstly investigated predicted compounds with strong negative and positive connectivity and aimed to identify support for their supposed activity in the scientific literature. Among the highly ranked compounds showing strong positive connectivity (correlated gene expression signatures), several compounds already have published evidence of the effect to be determined experimentally, namely Meglumine (rank 9 out of 6100), Troglitazone (rank 17) and *α*-Estradiol (rank 20). Meglumine is capable of differentiating bone marrow mesenchymal stem cells to cardiomyocytes,^20^ whereas Troglitazone induces heart cell proliferation.^21^ Estradiol demonstrates cardioprotective effect against cardiac injury^22^ and induces proliferation, but not differentiation, of undifferentiated myoblast cells. On the other hand Resveratrol (rank 2) and Pyrvinium (rank 29) show strong negative connectivity, and it has been established before that Resveratrol-modified cardiac stem cells regenerate infarcted myocardium.^23^ Pyrvinium, on the other hand, promotes wound repair and post-myocardial infarct cardiac remodeling.^24^ It also ameliorates myocardial contractile dysfunction in a mouse model of myocardial infarction.^25^ Overall significant literature support for the claimed effects could hence be identified in the first step of this study.

We next proceeded to select compounds for the prospective validation of the algorithm. Among compounds with positive connectivity Bethanechol (rank 2), Prilocaine (rank 4), Famotidine (rank 12) and Sodium Phenylbutyrate (rank 18, Butyrate) have been selected for prospective experimental validation owing to their high rank and novelty in the given context. The effect of treatment of these compounds on stem cell differentiation was investigated as presented in the next section.

### Enhancement of cardiomyocytes during CPC formation

**Figure 2:**
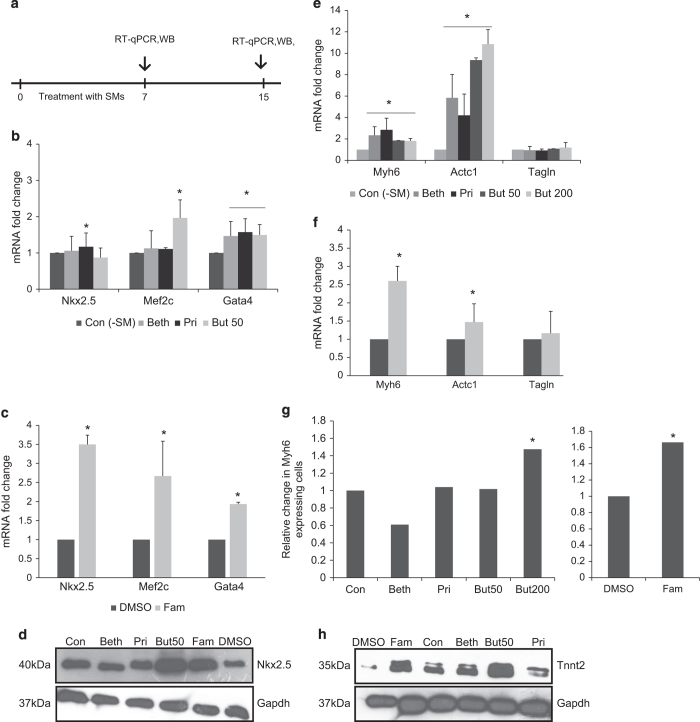
Cardiac differentiation efficiency during CPCs formation treated with predicted compounds. (**a**) Protocol of compound treatment of mESCs during cardiac differentiation, (**b** and **c**) RT-qPCR analysis for precursor markers, (**d**) western blot analysis for Nkx2.5, (**e** and **f**) RT-qPCR analysis for mature cardiomyocyte markers (*Actc1* and *Myh6*) and smooth muscle cells markers (*Tagln*), (**g**) percentage of cells expressing Myh6 protein identified by flow cytometry at day 15, (**h**) western blot analysis for Tnnt2. Relative expression of target genes was quantified and normalized with Gapdh. The number of independent repeats was three for each experiment (*n*=3). Value bars are mean±S.E.M.

To investigate the effect of Butyrate, Famotidine, Prilocaine and Bethanechol on cardiogenesis from mouse embryonic stem cells (mESCs) embryonic bodies (EBs) were treated with each individual compound from day 2 to 7 (see experimental procedures section for details) and cardiomyocyte differentiation was assessed both qualitatively and quantitatively (Supplementary Figures S1 and Figure 2).

Qualitative assessment was performed by comparing morphological features of the beating cells to controls. It was found that the treatment of EBs with all selected compounds, particularly Famotidine, increased their expansion rate of plated or beating EBs, as well as the degree of contraction (Supplementary Figures S1a and f; see also Supplementary Movies S1–S4 for details). As these data are observational and subjective, therefore, the main conclusion were based on quantitative assessment provide in the section below.

We next performed quantitative characterization of cardiac markers using quantitative reverse transcriptase PCR (RT-qPCR). On day 7, the expression of *Gata4*^26^ as mesoderm cardiac marker and *Nkx2*-5^27^ and *Mef2c*^28^ as cardiac precursor markers, all of which are required for cardiac differentiation and development, was examined. It was found that expression of *Gata4* (Figures 2b and c) was increased 1.49–1.93 folds by all compounds with the highest increase (of 1.93 folds) observed for Famotidine. Unlike in case of *Gata4*, increased expression of *Nkx2.5* at the RNA level was only observed for Famotidine, where expression was found to be significantly (3.5 folds) higher than for control. This marker was increased at the protein level in the presence of both Famotidine and Butyrate (Figure 2d). Significant Increase in expression of *Mef2c* was also observed for Butyrate (1.96 folds) and Famotidine (2.66 folds, Figures 2b and c).

**Figure 3:**
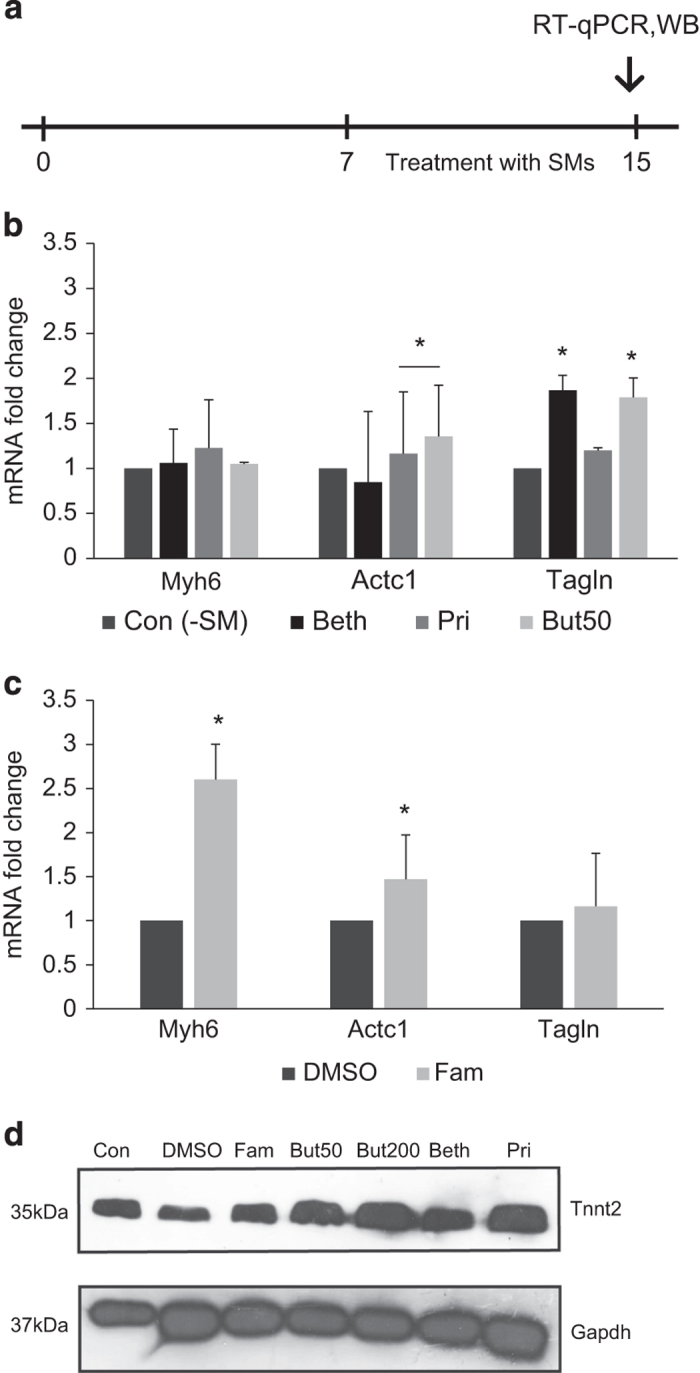
Alteration of transcript levels of precursor and mature markers post-CPCs formation. (**a**) Protocol for compound treatment of mESCs post-CPCs formation on EBs for 7 days, (**b** and **c**) RT-qPCR assessment of mature cardiac markers. Relative expression of target genes were quantified and normalized with Gapdh. The number of independent repeats was three for each experiment (*n*=3). Value bars are mean±S.E.M., (**d**) western blot analysis for Tnnt2 marker. Intracellular protein amounts were calculated relative to the Gapdh content.

On day 15 expression of *Actc1* and *Myh6* as cardiomyocytes-specific markers and *Tagln* as smooth muscle marker were examined also by RT-qPCR. The specific expression of the *Actc1* (*α*-actin) gene represents the start of differentiation of cardiac cells^29^ and it is expressed in adult heart.^30^ *Myh6* on the other hand is expressed in healthy, but not failing, human heart and has a major role in cardiac contractility.^31^ *Tagln* is expressed in the embryonic heart^32^ but its expression diminishes in adult heart.^33^ Treatment with Butyrate, Bethanechol, Prilocaine and Famotidine significantly enhanced mRNA levels of *Actc1* (by 9.3, 5.8, 4.1 and 1.47 folds, respectively) and *Myh6* (1.8, 2.3, 2.8 and 2.6 folds) on day 15 versus their respective controls, while levels of *Tagln* remained unchanged (Figures 2e and f). Immunostaining corroborated increased *Myh6* expression after treatment with Famotidine and Butyrate (Supplementary Figure S1g). Cells were furthermore examined on day 15 for Myh6 by flow cytometry in order to determine its expression at the protein level. Famotidine-treated cells resulted in cardiomyocytes with 56% of cells testing positive for the Myh6 protein, compared with 33% positive cells when treated with DMSO (1.7 times increase in purity; see Discussion section to put those numbers into context). Cardiomyocytes treated with 200*μ*M of Butyrate increased the amount of Myh6 protein positive cells from 39 to 58% (1.48 times increase in purity, Figure 2g). Moreover, the Tnnt2 protein level was assayed by western blot, with results being consistent with a differentiation of stem cells to cardiomyocytes (Figure 3h). We can hence overall conclude that Famotidine and Butyrate significantly enhanced cardiac differentiation efficiency versus DMSO (As famotidine was dissolved in DMSO, we had separate DMSO treatment for comparison) and control, respectively.

### Effects of compound treatment on post-CPC formation

In this stage, EBs were cultured on the gelatin coated adherent dishes in K-DMEM and 15% ES-FCS from day 7 to day 15. To investigate the effect of selected compounds after cardiac progenitor formation, these compounds were added to the medium in this stage (Figure 3a) and cells were examined for cardiac markers expression by RT-qPCR (Figure 3b). *Myh6* was upregulated significantly by Famotidine (2.6 folds, Figure 3c), but such an effect was not observed for the remainder of the compounds (Figure 3b). *Actc1* expression was stimulated by Famotidine (1.47 folds), whereas *Tagln* (smooth muscle marker) was overexpressed (1.7 and 1.8 folds) by Butyrate and Bethanechol treatment, respectively. Western bloting confirmed the expression of Tnnt2 as a mature cardiac marker for all of the compounds, particularly Prilocaine and Butyrate, at the protein level (Figure 3d). Hence, we can conclude overall the compound addition to the medium post-CPC formation (days 7 to 15) also enhances the differentiation to cardiomyocytes.

### Mode of action analysis of Famotidine in cardiac regeneration

We next investigated the potential mode-of-action of the best-performing compound, Famotidine, in order to understand its putative activity in more detail. Previously, it has been shown that Famotidine reduced infarct size of cardiomyocytes in mice after ischemia/reperfusion injury or permanent ischemia.^34^ These effects had been associated to the anti-histamine effects of Famotidine rather than its role in promotion of cardiac differentiation.^34^ On the basis of our analysis involving both *in silico* target prediction and the utilization of gene expression data about compound action (Figure 1) we would now suggest that this effect might be associated to multiple proteins and genes and pathways targeted by Famotidine involved in cellular differentiation, in addition to antagonizing the Histamine H2 receptor. Target prediction suggested that Famotidine targets the Multidrug and toxin extrusion protein 1, Complement C1s, Thrombin and the Histamine H2 receptor as well as the Urokinase-type plasminogen activator. Among these targets, Multidrug and toxin extrusion protein 1 and Histamine H2 receptor are supported directly by ChEMBL with experimental IC_50_ values of 760 and 42 nM, respectively. Famotidine is predicted to target Complement C1s protein, whose inhibition has previously been suggested to have therapeutic value for myocardial infarction.^35^ On the other hand, the third predicted target, thrombin, modulates phosphoinositide and calcium metabolism of the heart, increases beating rate, diastolic calcium and peak systolic calcium of spontaneously contracting cultured ventricular myocytes and improves automaticity and impulse initiation.^36^

When considering the gene expression changes induced by Famotidine, the GSEA algorithm was used to identify the most enriched upregulated genes in common between the embryonic stem cells *versus* heart (ESCvsHRT) signature and the set of the 50 most upregulated genes of Famotidine instance in CMap (instance ID 5011, applied on MCF7). These genes were found to be *GRPR*, *RHBG*, *FSCN1*, *FGFR3*, *KIAA1609*, *WIPI2*, *ST8SIA3*, *GLP1R*, *ING1*, *SLC24A1*, *CHRNA3* and *DNAJC16*. Among those genes particularly *FGFR3* and *GRPR* are of interest here, as fibroblast growth factors including *FGFR3* regulate epicardial signaling and myocardial growth and differentiation.^37^ *GRPR* on the other hand has a role in smooth muscle contraction.^38^ On the other hand the most enriched downregulated genes of the Famotidine instance, that are also downregulated in the ESCvsHRT signature, are *SIPA1L3*, *ROM1*, *HABP4*, *SLC25A23*, *SCRN3*, *CCL14*, *PLEKHO1*, *CTDSPL*, *SOS2*, *ZNF639*, *FSTL3*, *ARHGEF9* and *LRP6*. The Famotidine instance downregulated an essential Wnt co-receptor, LRP6, from the ‘Canonical Wnt signaling’ pathway which was also downregulated in the ESCvsHRT. Modulating the Wnt signaling pathway is known to have an important role in differentiation of stem cells to cardiomyocytes.^39^ The compound also downregulates Notch2 from the ‘Notch Signaling Pathway’, which was upregulated in the ESCvsHRT signature. Perturbation of the Notch Signaling Pathway regulates cardiac differentiation during development in mammals and also has an important role in recovery from adult myocardial damage.^39,40^

To summarize, the positive effect of Famotidine in ischemia seems to go far beyond its activity on the Histamine receptor alone, and by combining predicted protein targets and differentially expressed genes after compound application provides a far more unified picture of the mode-of-action of Famotidine in this context.

## Discussion

In this work, a general method for selecting small molecules for directed stem cell differentiation has been presented. To this end, large scale gene expression profiles of compound treatments (1309 compounds in CMap) were utilized, which were then matched with gene expression profiles of the particular target cell line of interest, compared with the cell line of origin (here and more generally in the area of stem cell differentiation, stem cells). This approach has then experimentally been validated by predicting compounds for promoting differentiation of stem cells to cardiomyocytes. Four compounds (Famotidine, Bethanechol, Prilocaine and Sodium Phenylbutyrate) were experimentally validated in this study for their ability to stimulate gene and protein levels of cardiac precursor and cardiac markers. Each of the four compounds stimulated the markers in agreement with the hypothesis. However, Famotidine and Butyrate had more significant effect on the cardiac and cardiac progenitor markers and hence had superior effect on the differentiated cardiomyocytes. Famotidine and Butyrate increased the percentage of Myh6-positive cells from 33% and 39%, respectively, to 56% and 58%, respectively, compared with control groups (DMSO treatment, no treatment). To put those numbers into context, a recently discovered small molecule for cardiomyocyte differentiation, Zebularine, increased the percentage of Myh7-protein-positive cells (also responsible for cardiac contractile function) from 27 to 35% (an 1.29-fold increase), so at considerably lower levels.^7^ Famotidine is predicted to act by inhibiting of Complement C1s and thrombin at the protein level, and by upregulating *GRPR and FGFR3*, as well as downregulating LRP6 and Notch2 on the gene level, which gives a much more unified picture of compounds action both from the ligand-target interaction level and the genetic level than previous studies have found.

Overall, the computational approach proposed here shows that gene expression data, in combination with *in* silico target prediction, can be used for the selection of small molecules to promote the targeted differentiation of embryonic stem cells to cardiomyocytes, as well as suggesting possible mechanism of actions of the small molecule. Given the general nature of the approach it is expected that this method can be extended to the selection of small molecules for promotion of stem cell differentiation to any tissue of interest.

## Materials and Methods

### Compound prediction approach

For identifying compounds that are anticorrelated with the heart signature GSEA as implemented by the Broad Institute^17^ was employed in this work. This method checks whether a query gene signature is occurring at the extremes (top or bottom) of a rank-ordered list of genes, or whether it shows closer to random distribution (ie., no correlation between both spaces). An enrichment score was calculated by descending the rank-ordered list of genes and incrementing a variable when encountering a gene in the given query signature. The magnitude of increment depends on the position of the gene in the list, which is chosen corresponding to a weighted Kolmogorov–Smirnov statistic. The enrichment score can hence range between −1 and 1, where −1 shows strong negative connectivity, 1 identifies strong positive connectivity and 0 represents zero connectivity. In our particular case, the signature of each compound from CMap was used to query rank-ordered list of genes of a disease and both positive and negative connectivities were analyzed subsequently.

The input of the GSEA algorithm is a query gene signature, a gene expression profile (gct), a phenotype annotation file (cls), and a probeset to gene symbol translation file (chip). A further parameter is the number of top and bottom genes of the rank-ordered list of compound gene expression profile to be selected, where in the current study this was selected to be 50. In this list the ranking of genes was based on log2 ratio of the treatment of compound to vehicle. Generally, how many genes to select is based as much on intuition as on rational criteria, and in our experience the best number of genes to be selected generally varied between those numbers. This left us with two query signatures, namely one of the most upregulated genes, and one of the most downregulated genes. For each disease *versus* drug the two signatures were used to query the disease profile, hence giving rise to two scores, the ‘up’ score and the ‘down’ score. The following formula was used to combine the two scores into one score:

$$\mathrm{score}=\frac{{\mathrm{score}}_{\mathrm{up}}-{\mathrm{score}}_{\mathrm{down}}}{2}$$

In this formula score_up_ and score_down_ are the scores calculated for the compound given its most x up- and downregulated genes, respectively. In the GSEA results a negative score means that the query signature was found at the bottom of the list and a positive score means that the query signature was found at the top of the list. The combined score was used to sort the compounds and come up with a rank-ordered list.

### Annotation of the disease relevance of genes and proteins via CTD

Although gene expression data are able to map compound and disease spaces with the parameters chosen, we also employed computational tools to hypothesize protein target-based bioactivity spectra for selected compounds, and to evaluate the mode-of-action of those (and their potential novelty), based on additional database information we integrated. For this purpose, the Comparative Toxicogenomics Database (CTD)^19^ has been used to identify genes that are associated with cardiovascular diseases, where disease–gene links were downloaded separately as provided on the CTD website. The CTD database provides an Inference score for each target, which indicates the level of relevance of each target with cardiovascular diseases, based on text mining approaches of a large set of scientific publications. The gene–protein links were retrieved from ChEMBL^41^ to map gene identifiers to proteins implicated in diseases.

### *In silico* target prediction to support compound selection and mode-of-action analysis

A target prediction algorithm as established before^18^ has been utilized to predict protein targets of compounds in the CMap databases using the Naïve Bayes approach. This algorithm predicts a score for each protein target included in the training set, which represents the probability of the compound to bind to this target (without considering functional effects). The extraction of compound-target pairs was identical to the benchmarking data set query introduced in the previous work^18^ (which included targets with binding affinity <10 *μ*M and confidence level of 9 or 10) except that it was applied on ChEMBL^41^ v.17 and hence left us with a larger training database of 385 126 compound-protein pairs, 1643 distinct proteins and 226 791 unique compounds. Compounds were standardized and ECFP4 fingerprints were generated using the JChem package of ChemAxon.^42^ The standardization options were Aromatize, RemoveExplicitH, Clean 2D, Clean 3D, RemoveFragment and Neutralize. The Laplacian modified Naïve Bayes version of the algorithm provided in the previous publication^18^ was then trained on the extracted.

The protein target predictions for all compounds to be analyzed (in this case all compounds from CMap) was incorporated with the gene expression-based results and the whole information is then displayed to the user, enabling the selection of compounds based on connectivity derived from gene expression profiles, predicted bioactivity spectra (modes-of-action), as well as relevance of each target for the disease. ChEMBL Therapeutic Flags of compounds have been used to check whether the drug is approved.

### Culture method and differentiation of MESCs to beating cardiomyocyets

Cell culture chemicals were supplied by Life Technologies (Grand Island, NY, USA) unless indicated otherwise. Mouse embryonic stem cells strain RB20 from C57BL/6 strain was used as reported. Twenty cells cultured in serum-free media, Dulbecco’s modified eagle medium F12 (DMEM F12) and Neurobasal media with 0.5% BSA, 2 mM glutamine, 0.1 mM nonessential amino acids, 1% penicillin–streptomycin (all from Invitrogen, Shanghai, China), 0.1 mM *β*-mercaptoethanol (Sigma-Aldrich, St. Louis, MO, USA) and 1000 U/ml leukemia inhibitory factor (LIF; Chemicon, Temecula, CA, USA), 1 *μ*m PD0325901(Sigma-Aldrich) and 10 *μ*m SB431542 (Sigma-Aldrich), 1% N2 and 2% B27 (3).

The media used for cardiac differentiation consisted of K-DMEM, supplemented with 15% ES-FCS, 0.1 mM nonessential amino acids, 2 mM L-glutamine, 0.1 mM beta-mercaptoethanol, 1% penicillin–streptomycin and ascorbic acid. For cardiac differentiation, EBs were formed by adding 800 ESCs per hanging drop over two consecutive days (day 2). Following collection of EBs, they were cultured in suspension for the next five days (day 7). On the seventh day, the EBs were plated on gelatin coated 12-well plates (Techno Plastic Products, Trasadingen, Switzerland) for further 6 to 8 days (day13–15).^43^

To obtain the optimal concentration of each predicated compound, the concentration predicated based on pervious literature was taken as the bases and lower and higher concentration was added during EB formation and the concentrations which reduced the EB size were taken as toxic concentration and the lower concentrations which had no toxic effect was selected for further experiments. Therefore, the selected concentrations for Butyrate, Famotidine, Prilocaine and Bethanechol were 50, 10, 15 and 20 *μ*M, respectively. 200 *μ*M of Butyrate was also used where indicated in results.

### Effect of the predicted compounds on cardiac precursor formation and cardiac differentiation

To evaluate the effect of predicted compounds on cardiac precursor formation, the compounds were added during the first 5 days of EB formation (day 2–7), whereas for cardiac differentiation, the compounds were added during plating (day 7–15). All the compounds were dissolved in water, except for Famotidine, which was dissolved in dimethyl sulfoxide (DMSO). Therefore, equal amount of the DMSO was also added to the control of this group. To evaluate the effect of these compounds on cardiac precursor formation and cardiac differentiation, the cells were evaluated microscopically for morphology, by RT-qPCR for gene expression, by western blot for protein expression and by flow cytometry for efficiency of cardiac differentiation.

### Quantitative reverse transcriptase PCR analysis

RNA was extracted using RNeasy Mini Kit (Qiagen, Hilden, Germany) and cDNA was made using Moloney murine leukemia virus reverse transcriptase, 1 *μ*g of each RNA sample, and random hexamer primers according to the manufacturer’s protocol. Reverse transcription quantitative real-time PCR (RT-qPCR) was implemented by gene specific primers and SYBR Green (TaKaRa, Otsu, Japan). Applied Biosystems step one plus Real-Time PCR system (Foster, CA, USA) was used for Real time PCR analyses. For each reaction in a final volume of 10 *μ*l, the PCR mixture contained 5 *μ*l Rotor-Gene SYBR Green PCR Master Mix, 3 pmole of each primer, and 25 ng of cDNA. The relative mRNA concentrations were calculated using the software provided by the manufacturer. In order to normalize the data, Gapdh expression level was measured in the same sample. All measurements were carried out in triplicates, and the data was assessed and reported according to 2^−ΔΔCt^ method. Specific primer pairs were designed by the Beacon designer (Version 7.2, Premier Biosoft International, Palo Alto, CA, USA) and Perl-primer ordered through metabion company (Martinsried, Germany).

### Immunocytochemistry analysis

An indirect immunofluorescence light microscopy was applied to analyze the cells as previously described.^43^ The primary antibodies were anti-mouse antibodies against Myosin Heavy Chain (Myh6, 1 : 300, Abcam, Cambridge, UK). The secondary antibody was, fluorescein isothiocyanate (FITC)-goat anti-mouse IgG (1 : 50, Chemicon). Meanwhile, the nuclei were counterstained with DAPI. The stained cells were analyzed with a fluorescent microscope (Olympus, Tokyo, Japan) and images were acquired with an Olympus DP70 camera (Olympus, Tokyo, Japan).

### Western blot analysis

Cells were lysed with TRI reagent (Sigma-Aldrich) according to the manufacturer’s protocol. Thirty *μ*g of solubilized protein fraction of each sample was subjected to SDS-PAGE electrophoresis and transferred to Polyvinylidene fluoride (PVDF) membrane. The samples were blocked on the membrane by adding 5% skim milk and the respective bands were labeled with mouse anti Tnnt2 antibody (Abcam, ab8295, dilution: 1/5000), rabbit anti NKX2.5 antibody (Santa Cruz Biotechnology, Santa Cruz, CA, USA, Sc14033, dilution: 1/2000) and mouse anti GAPDH antibody (Santa Cruz Biotechnology, dilution: 1/5000). The secondary antibodies were horseradish peroxidase (HRP)-conjugated goat anti-mouse IgG (DAKO, Carpinteria, CA, USA) and HRP-conjugated goat anti-rabbit IgG (Santa Cruz Biotechnology). HRP-conjugated IgG bound to each protein band was visualized by an Amersham ECL Advance Western Blotting Detection kit (GE Healthcare, Madison, WI, USA).

### Flow cytometry analysis

In order to estimate the content of Myh6 in cardiac cells, cells were fixed with 4% paraformaldehyde, and permeabilized by treatment with Triton X-100 at a final concentration of 0.4% (V/V). Treated cells were incubated with Myh6 antibody for 1h at 37 °C, and subsequently were labeled with goat fluorescence isothiocyanate (FITC) anti-mouse as a secondary antibody for 40 min at 37 °C. The fluorescence intensity of cells, which represented Myh6 expression levels were analyzed by a Becton Dickinson FACSCalibur flow cytometer (Becton Dickinson Biosciences, San Jose, CA, USA). For each sample, 104 events were recorded in the forward light scatter/side light scatter (FSC/SSC) dot plot. Both green fluorescence FITC for Myh6 were detected in a fluorescence detector 1 (FL-1) with a 530/30 nm band pass filter. Data were analyzed by comparison of the mouse IgG1 negative isotype control (final concentration, 1 : 200).

### Statistical analysis

SPSS (version 17, IBM SPSS Statistics, Chicago, IL, USA) was used to present data as means±SEM obtained from three independent treatments of the replicated observations. One-way analysis of variance (ANOVA) was applied to identify the statistical differences between the three or more treatments. Also independent t-test analysis was carried out to identify statistical differences between the two treatments. The threshold of *P*<0.05 (*) was used to indicate levels of statistical significance.

## Abbreviations

GEO: Gene Expression Omnibus
CMap: Connectivity Map
CTD: Comparative Toxicogenomics Database
CPC: cardiac progenitor cells
mESCs: mouse embryonic stem cells
EBs: embryonic bodies
RT-qPCR: Quantitative reverse transcriptase PCR
ESCvsHRT: embryonic stem cells *versus* heart
GSEA: Gene Set Enrichment Analysis
ESCs: embryonic stem cells
DMSO: dimethyl sulfoxide
PVDF: polyvinylidene fluoride.
