# Supplementary figures and images for: Systematic selection of small molecules to promote differentiation of embryonic stem cells and experimental validation for generating cardiomyocytes
Source: Cell Death Discov. 2016 Feb 8;2:16007–. doi: 10.1038/cddiscovery.2016.7 (PMC4979408; doi:10.1038/cddiscovery.2016.7)

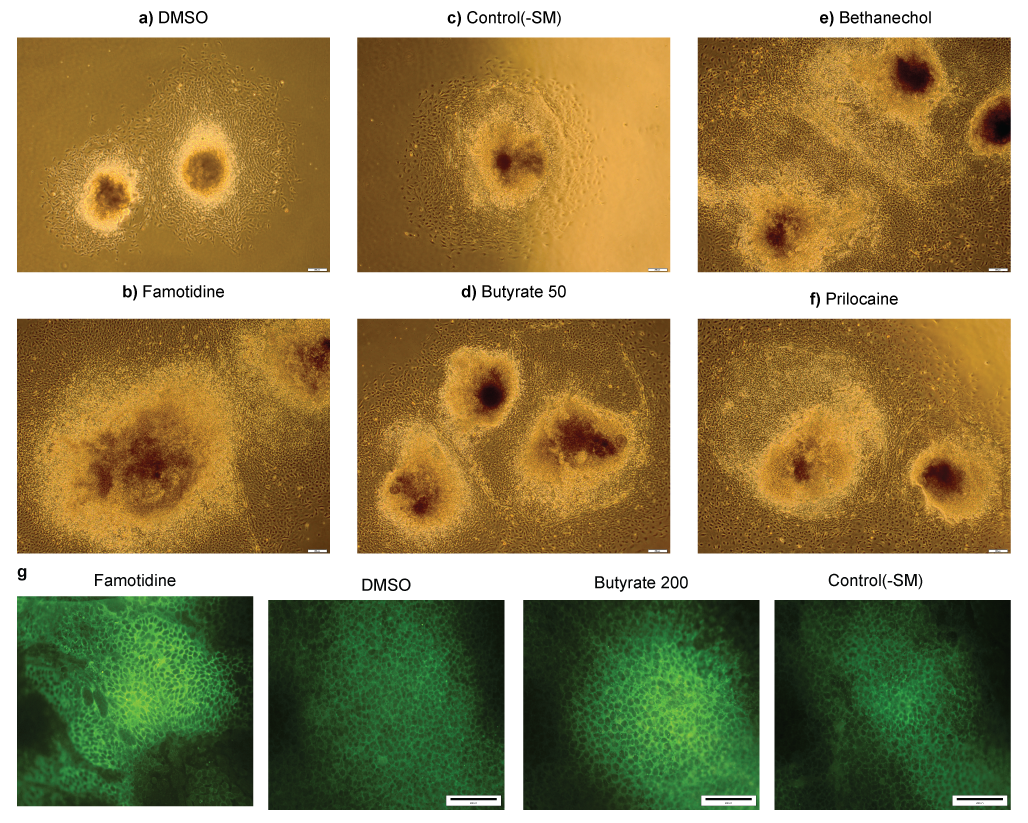

Supplement: Supplementary Figure S1 [file cddiscovery20167-s1.tiff]
